# ACSwinNet: A Deep Learning-Based Rigid Registration Method for Head-Neck CT-CBCT Images in Image-Guided Radiotherapy

**DOI:** 10.3390/s24165447

**Published:** 2024-08-22

**Authors:** Kuankuan Peng, Danyu Zhou, Kaiwen Sun, Junfeng Wang, Jianchun Deng, Shihua Gong

**Affiliations:** 1 Digital Manufacturing Equipment and Technology Key National Laboratories, Huazhong University of Science and Technology, Wuhan 430074, China; d202080257@hust.edu.cn (K.P.); d202280332@hust.edu.cn (D.Z.); kevinsun101@hust.edu.cn (K.S.); dengjianchun@hust.edu.cn (J.D.); 2 Huagong Manufacturing Equipment Digital National Engineering Center Co., Ltd., Wuhan 430074, China; 3 Tongji Hospital, Tongji Medical College, Huazhong University of Science and Technology, Wuhan 430030, China

**Keywords:** Swin Transformer, anatomical constraint, perceptual similarity, CT-CBCT rigid registration, IGRT

## Abstract

Accurate and precise rigid registration between head-neck computed tomography (CT) and cone-beam computed tomography (CBCT) images is crucial for correcting setup errors in image-guided radiotherapy (IGRT) for head and neck tumors. However, conventional registration methods that treat the head and neck as a single entity may not achieve the necessary accuracy for the head region, which is particularly sensitive to radiation in radiotherapy. We propose ACSwinNet, a deep learning-based method for head-neck CT-CBCT rigid registration, which aims to enhance the registration precision in the head region. Our approach integrates an anatomical constraint encoder with anatomical segmentations of tissues and organs to enhance the accuracy of rigid registration in the head region. We also employ a Swin Transformer-based network for registration in cases with large initial misalignment and a perceptual similarity metric network to address intensity discrepancies and artifacts between the CT and CBCT images. We validate the proposed method using a head-neck CT-CBCT dataset acquired from clinical patients. Compared with the conventional rigid method, our method exhibits lower target registration error (TRE) for landmarks in the head region (reduced from 2.14 ± 0.45 mm to 1.82 ± 0.39 mm), higher dice similarity coefficient (DSC) (increased from 0.743 ± 0.051 to 0.755 ± 0.053), and higher structural similarity index (increased from 0.854 ± 0.044 to 0.870 ± 0.043). Our proposed method effectively addresses the challenge of low registration accuracy in the head region, which has been a limitation of conventional methods. This demonstrates significant potential in improving the accuracy of IGRT for head and neck tumors.

## 1. Introduction

**Figure 1:**
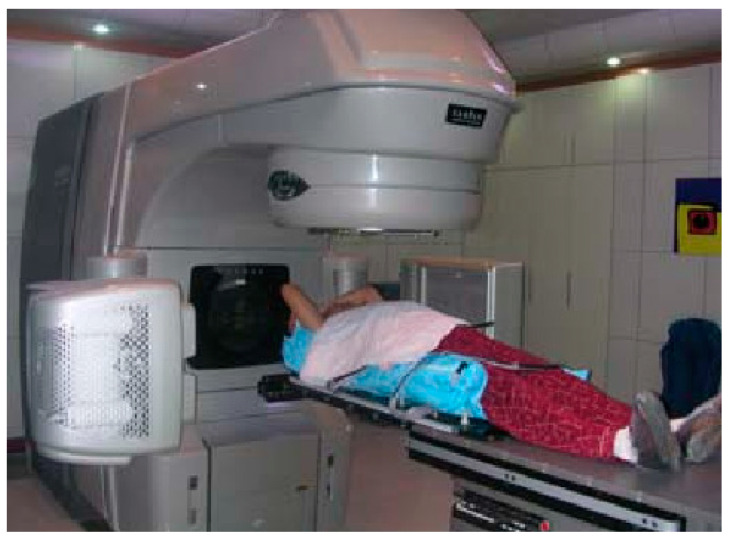
Image-guided radiotherapy system.

Head and neck cancer, characterized by a high number of lesions and complex distribution, is among the most lethal types of tumors in the human body [1]. Radiotherapy is a prevalent treatment for malignant tumors, but setup errors in the treatment couch position remain a significant obstacle to achieving precise radiotherapy [2]. IGRT has emerged as a promising technique to mitigate setup errors and enhance treatment accuracy. CBCT is frequently used in IGRT to obtain volumetric images of patients. As shown in Figure 1, the CBCT flat panel detector rotates around the patient once to acquire the volume image of the patient [3]. During IGRT, CBCT images are rigidly registered with planning CT images to guide the treatment couch and correct any deviations so that the organs, tissues, and target regions identified in the planning CT are aligned with the current radiotherapy position of the patient. This alignment allows for accurate dose delivery to the target region, minimizing harm to normal organs and tissues. Therefore, precise rigid registration between CT and CBCT images is vital for accurate radiotherapy, protecting adjacent normal tissues from radiation injury, and reducing complications [4,5].

Currently, conventional image registration methods are predominantly employed for CT-CBCT rigid registration in clinical practice. These methods define an image similarity metric that assesses how closely the moving image matches the fixed image as the optimization objective function, and then optimize the objective function by iteratively updating the rotation translation matrix parameters. However, conventional multimodal registration similarity metrics, such as cross-correlation(CC) [6], mutual information (MI) [7], and normalized mutual information(NMI) [8,9], may not be well-suited for CT-CBCT registration. This is due to the varying intensity of artifacts in CBCT images, making the establishment of correspondence between CT and CBCT images challenging [10,11]. Moreover, the iterative process can be time consuming [12], especially when there is significant initial misalignment between the two images, leading to increased processing time. Even more unfavorable is the propensity to become stuck in local optima [13], thus failing to meet clinical demands.

In contrast, deep learning-based rigid registration methods can employ various techniques to measure the similarity between images, aligning the moving image to the fixed image in one step by learning the direct correspondence between input image pairs, enhancing registration accuracy while also reducing the time required for iteration [14]. According to the literature [15], approximately 19% of the relevant papers involve research on deep learning-based rigid registration. Although this proportion is lower compared with papers focusing on non-rigid registration (72%), the field continues to receive sustained attention and research. As early as 2015, Miao et al. [16] demonstrated the efficacy of deep learning in rigid registration. Miao et al. employed convolutional neural networks (CNNs) to achieve real-time rigid registration of digitally reconstructed radiographic images and X-ray images. Guo et al. [17] developed a cascaded CNN network for coarse-to-fine multi-stage rigid registration of magnetic resonance images (MRI) and ultrasound images (USI). When the initial error is 8 mm, the method improves the registration accuracy from 6.42 mm with conventional methods to 3.57 mm. Salehi et al. [18] employed deep learning methods to achieve real-time 3D pose estimation for rigid registration, significantly enhancing the real-time performance and accuracy of medical image registration. The method achieves 3D pose estimation in just 100 ms, outpacing conventional methods by 25 to 40 times and improving tolerance to initial misalignments. While conventional methods fail beyond 80-degree image orientations, with errors up to 180 mm, the deep learning approach maintains accuracy below 21 mm. This is of great significance for improving the accuracy and efficiency of radiotherapy. Deng et at. [19] designed an interpretable universal framework for both rigid and non-rigid multimodal image registration. In this model, the multi-modal features that are affecting the image alignment are well separated from the features that are not useful for registration. The effectiveness of the model is evaluated on natural images and brain MRI images.

However, according to the literature [20], research on head and neck registration is notably limited, comprising a mere 2% of the entirety of deep learning-based medical image registration studies. Thus, the deep learning-based registration method for head and neck regions requires further research and exploration. Most deep learning-based registration methods adopt CNN as their architecture [21,22,23,24]. Although CNN has achieved great success in many registration tasks, a recent study [25] has highlighted the poor performance of CNN in seemingly simple coordinate transformation tasks. In a simple supervised coordinate classification task designed by the authors, the accuracy rate was found to be only 86%. This might be because, while CNN is better at understanding local information, it usually has limitations in comprehending remote spatial relationships [26] (i.e., the relationship between two pixels that are far apart). Rigid image registration, on the other hand, requires global transformation, which suggests that the CNN architecture is not an ideal choice for handling rigid registration tasks.

To address this limitation, researchers proposed the Vision Transformer [27], which is notable for its long-range modeling capabilities. Experiments have demonstrated that it performs remarkably in image recognition, segmentation and detection. ViT-V-Net [28] first applied the Transformer to the domain of image registration. ViT-V-Net mixes CNN and Transformer architectures and outperforms pure CNN-based methods. It achieved a Dice score that is 1.5 percentage points higher than VoxelMorph on the brain MR dataset, reaching 0.726. Tony C.W.Mok et al. [29] proposed a cascaded Transformer network to perform affine registration on the image from coarse to fine. This method improves the atlas-based MRI registration accuracy from a DSC of 0.67 with conventional methods to 0.69, while also achieving a registration speed over 50 times faster. Chen et al. [30] proposed Transformer-based registration method TransMatch. This explicitly matches multilevel features between image pairs, demonstrating its effectiveness in establishing accurate inter-image correspondences. Although Transformer is remarkable in modeling long-range dependencies, it has quadratic computation complexity to the image size, which is time-consuming.

**Figure 2:**
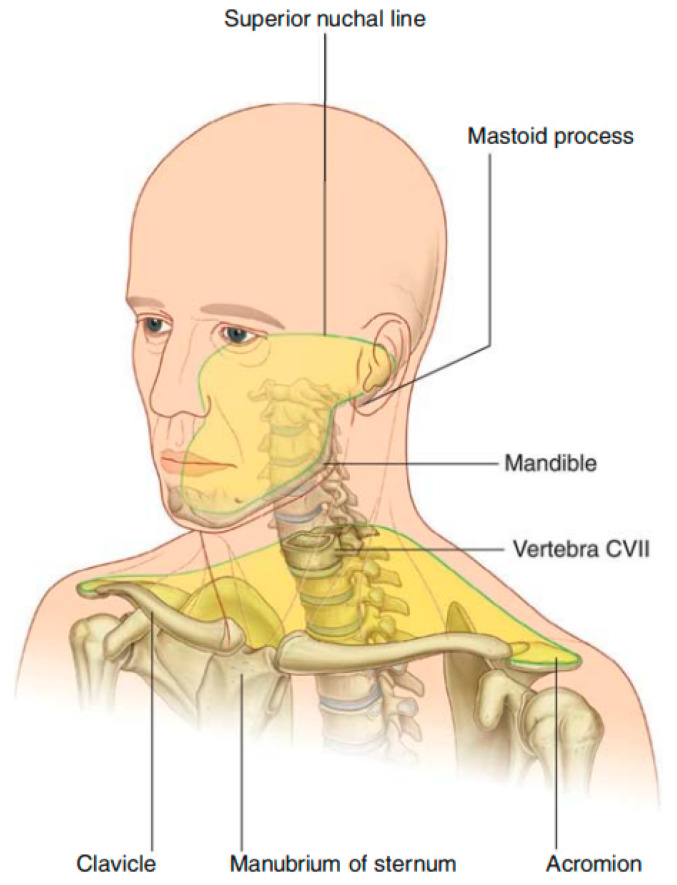
Boundaries of the neck.

Another issue that arises during the rigid registration of the head and neck is the occurrence of minor non-rigid deformations due to changes in patient positioning. This can result in rigid registration failing to ensure alignment for all regions. Radiotherapy can only be administered when all tumor regions meet the registration requirements. Figure 2 illustrates the boundary between the head and neck regions (superior nuchal line) [31]. In cases where the tumor extends widely across both the head and neck regions, the anatomical relationships among vital organs and tissues in the head region, such as the brainstem and optic nerves, are more intricate and concentrated and thus also more sensitive to dosage [32]. Therefore, a higher level of registration accuracy is necessary for the head region compared with the neck. However, current methods primarily adopt a global matching strategy, considering the head and neck as a single entity. This approach fails to recognize the increased need for precision in the head region. Consequently, while maintaining the same registration accuracy across both regions, the neck region may meet the standards, but the head region may fall short. To enhance the registration accuracy specifically in the head region, radiologists employ a local registration method that focuses solely on aligning the head region. While this approach can significantly improve the accuracy within the head region, it may simultaneously lead to substantially larger registration errors in the non-selected areas. Consequently, radiologists may be required to manually adjust the image pairs or even revise the patient’s radiotherapy plan, both of which are time consuming and laborious tasks. To our knowledge, current research has yet to focus on improving the accuracy of specific local regions within deep learning-based rigid registration applications.

Despite this, some methods mentioned in previous literature may inspire the addressing of this issue. Balakrishnan et al. [33] examined the impact of adding auxiliary information of anatomical segmentations on the accuracy obtained when registering brain MR images. With the auxiliary information, the method shows an approximately 0.03 improvement in DSC value, achieving 0.78 compared with VoxelMorph without the auxiliary information. The study demonstrated that adding anatomical segmentation information can effectively enhance the accuracy of image registration. Furthermore, it was observed that, when the segmentation maps of certain structures of the brain were incorporated into the network, the corresponding structures’ Dice coefficient improved. Mansilla et al. [34] have demonstrated that only pixel-level constraints on anatomical segmentations cannot guarantee good matching between moving images and fixed images on the global scale. Therefore, a pretrained encoder is used to encode anatomical segmentation into low-dimensional information to capture global information. As a result, this approach enhanced the DSC value of the outcomes by 0.02 across the three lung datasets, achieving a score of 0.93. The aforementioned models, which employ anatomical segmentations as auxiliary information to guide registration, have achieved significant improvements in various aspects. In our head-neck CT-CBCT rigid registration task, we adopt this approach, prompting the model to focus more on the head region while maintaining overall accuracy, thereby making the model more specialized in head-neck image registration.

To address the aforementioned issues, we present a deep learning-based rigid registration method. This method not only enhances registration accuracy and efficiency but also prioritizes the head region during the registration process. We utilize the Swin Transformer [35] as the backbone of our registration network in order to overcome the limitations of CNNs, specifically their small receptive fields, and effectively handle significant initial misalignments. To illustrate the benefits of our Swin Transformer-based registration network, we conduct a comparative analysis with two CNN-based networks: AC-VTN and AC-ConvNet. In addition, we introduce a perceptual similarity loss to evaluate the similarity between CT and CBCT images. This loss function utilizes a pretrained similarity measurement network to extract and analyze multi-scale hidden features. To meet the need for heightened registration accuracy in the head region, we incorporate an anatomical constraint network. This network leverages anatomical segmentation information of the head to guide the registration network to prioritize the head region, ultimately enhancing registration accuracy. Due to the dependence on anatomical segmentation information, the method proposed in the present study is a weak supervision method. It is worth noting that, after the model training is completed, the segmentation maps are no longer needed. Our method with ACSwinNet and other related deep learning-based registration methods are listed in Table 1.

In summary, the present study presents several significant contributions, as follows:

We introduce anatomical segmentation information into the rigid registration model for the first time, aiming to enhance the registration accuracy specifically in the head region for head-neck CT-CBCT rigid registration tasks. This issue has not been adequately studied before.

We propose a Swin Transformer-based rigid registration network to handle the large deformation between CT and CBCT images. The results of our experiments prove that the Swin Transformer-based network outperforms the CNN-based network.

Finally, we trained a perceptual metric network to analyze the differences in deep multi-scale features between CT and CBCT images. We used these differences as a loss function to guide the network training. Our code is freely available at https://github.com/pksolar/ACSwinNet (accessed on 15 May 2024).

## 2. Materials and Methods

The object of image registration is to align a moving image (denoted as *M*) to a fixed image (denoted as *F*) in n-dimensional space R^n^. In the present study, the CBCT image and its corresponding anatomical segmentation images are taken as moving images *M* and $S_{M}$, the planning CT image and its corresponding anatomical segmentation images are taken as fixed images *F* and $S_{F}$, and the CBCT image *M* is registered to the CT image *F* through rotation and translation in three-dimensional space. Various modeling methods are available for image registration, many of which treat it as an optimization problem. The purpose of rigid registration in the present study is to derive the rotation translation matrix through the registration network G(F,M)=A. After transformation by matrix *A*, the moving images *M* and $S_{M}$ are denoted as M(ϕ(A)) and $S_{M}(\phi (A))$ which makes them as similar to *F* and $S_{F}$ as possible. This process can be mathematically expressed as follows:

(1) $$A=\arg\min[D(F,M(\phi (A)))+L(S_{F},S_{M}(\phi (A)))]$$

where ϕ(A) is the deformation field corresponding to the transformation matrix *A*, and *D* is the dissimilarity metric between the CT images and the transformed CBCT images. This metric is implemented by a perceptual similarity metric network that has been pretrained to extract and analyze the deep features of the CT images and the transformed CBCT images. *L* is the dissimilarity metric for CT and CBCT anatomical segmentations, which is a conventional similarity measurement function and will be described in detail later. The sum of the two dissimilarity metric values represents the loss value for backpropagation, which is used to update the parameters of the registration network *G*.

**Figure 3:**
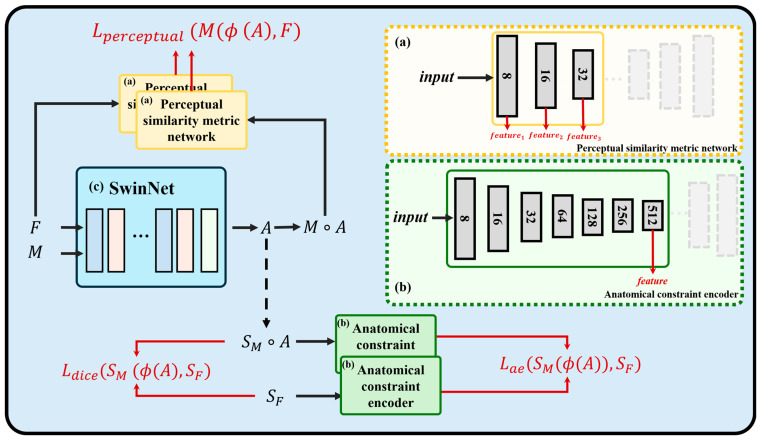
The architecture of the proposed Swin Transformer-based neural network with anatomical constraint for head-neck CT-CBCT images (ACSwinNet). The network incorporates a registration network based on Swin Transformer, while also including local constraint terms *L_dice_* and global constraint terms *L_ae_*. (**a**) The similarity metric network is used to calculate the perceptual loss between the fixed image and the transformed image (more detailed description is provided in Section 2.2). (**b**) The anatomical constraint encoder is used to encode the global information of the segmentation map. (**c**) The registration network SwinNet is used to generate the transformation matrix *A*. It should be noted that the similarity metric network and the anatomical constraint encoder have been pretrained through a denoising autoencoder network (a more detailed description is provided in Section 2.2). During the training of the registration network SwinNet, the weights of these two components remain fixed. In the inference phase of the model, the similarity metric network, the anatomical constraint encoder, and the segmentation maps are no longer required.

The overview of the proposed method is shown in Figure 3. The CT and the corresponding CBCT image with a size of 240 × 240 × 64 serve as input to the registration network *G*, which outputs a vector representing geometric transformation parameters instead of the transformation matrix, so that the problem can be simplified as G(F,M)=[t,r], where $t,r\in {\mathbb{R}}^{3}$ represents the translation and rotation parameters in the three-dimensional space. Once the rotation and translation parameters are known, the final rigid transformation matrix *A* can be obtained by matrix multiplication of translation matrix *T* and rotation matrix *R*, denoted as A=T·R, where *T* and *R* are derived by the corresponding *t* and *r*, respectively. The deformation field is then applied to transform the original CBCT image and CBCT anatomical segmentation, respectively. In Figure 3a, a pretrained 3-layer convolutional neural network is presented, which takes the transformed CBCT image and CT image as inputs and outputs multi-scale feature maps for calculating the perceptual loss. In part (b), the pretrained encoder takes the transformed CBCT segmentation map and CT segmentation map as input and outputs the encoded vectors for calculating the *L_ae_* loss.

### 2.1. Swin Transformer-Based Registration Network

**Figure 4:**
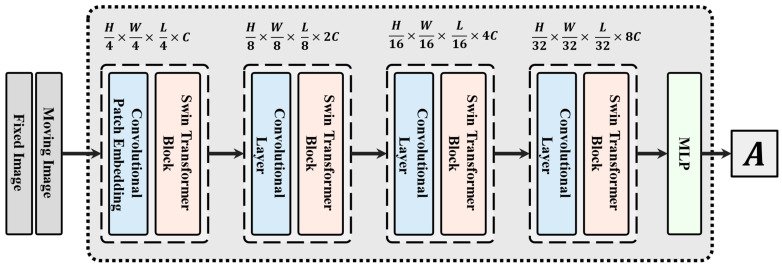
The architecture of Swin Transformer-based registration network. The network consists of four stages and is followed by a multilayer perceptron (MLP) module to generate the transformation parameters. Each stage is constructed of a convolutional layer and a Swin Transformer block. The output denoted as A signifies the parameters of the transformation matrix.

The proposed registration network structure is shown in Figure 4. Firstly, the CT and CBCT images with the size of H×W×L are concatenated to obtain the input I with the size of H×W×L×2. This input is then passed through a convolutional patch embedding layer with a convolutional kernel size of 7 and a stride of 4, resulting in an embedded feature with a size of $\frac{H}{4}\times \frac{W}{4}\times \frac{L}{4}\times C$. The registration network is composed of four stages, each of which contains a convolution layer and a Swin Transformer block. Except for the first stage, the convolution kernel of the convolution layer is 3, which results in a reduction in feature resolution by a factor of 2 and a doubling of the number of channels. The Swin Transformer block does not change the size of the feature map, so the output feature size at stage i is $\frac{H}{2^{i+1}}\times \frac{W}{2^{i+1}}\times \frac{L}{2^{i+1}}\times 2^{i-1}C$. After passing through the four stages, the features are fed into two fully connected layers for dimension reduction and the rotation and translation parameters are output.

**Figure 5:**
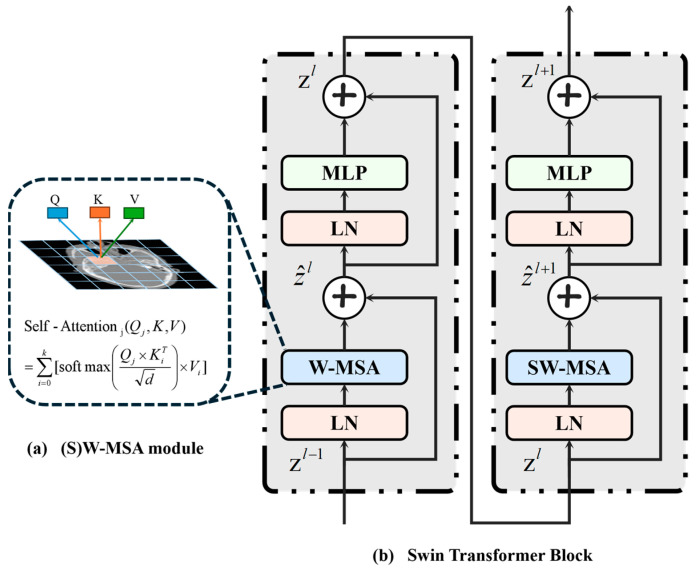
Swin Transformer block structure. (**a**) The self-attention mechanism is implemented in the (S)W-MSA module. (**b**) W-MSA and SW-MSA modules are embedded in the two successive Transformer blocks, respectively.

The utilization of a Swin Transformer block in the registration network is instrumental in facilitating the network’s understanding of a vast range of misalignment information between images. Illustrated in Figure 5, Swin Transformer is characterized by its window-based multi-head self-attention (W-MSA) and shifted window-based multi-head self-attention (SW-MSA) modules. These two modules replace the original MSA in two consecutive Transformer blocks. Different from computing the attention score between image patches of the entire image, window attention only calculates the attention score between image blocks within the same window, resulting in a significant reduction in computation. In addition, SW-MSA establishes a new window location, which consolidates image patches of different windows into the same new window, allowing for attention score computation across windows. This results in an expansion of the receptive field of the algorithm that is not restricted to the size of a single window. In addition, Swin Transformer has also devised a hierarchical structure and constructed a hierarchical representation of features through four stages and via the down-sampling operation between stages, making it flexible enough to model at various scales. The Swin Transformer block can be expressed by the following formula:

(2) $${\hat{z}}^{l}=W-MSA(LN(z^{l-1}))+z^{l-1},$$

(3) $$z^{l}=MLP(LN({\hat{z}}^{l}))+{\hat{z}}^{l},$$

(4) $${\hat{z}}^{l+1}=SW-MSA(LN(z^{l}))+z^{l},$$

(5) $$z^{l+1}=MLP(LN({\hat{z}}^{l+1}))+{\hat{z}}^{l+1},$$

where ${\hat{z}}^{l}$ and $z^{l}$ are the outputs of the (S)W-MSA module and the MLP module for the l block, respectively. Following the self-attention mechanism [36], the attention operation is computed as follows:

(6) $$Self-Attention(Q,K,V)=SoftMax(\frac{QK^{T}}{\sqrt{d}})V,$$

where *Q*, *K* and *V* represent the query, key and value matrices of the image patches, respectively, and *d* is the dimension of the query and key. The equation in Figure 5 is consistent with Equation (6). Figure 5a shows the calculation of the self-attention of the *j*-th patch.

### 2.2. Perceptual Similarity Metric Network

In the present study, the conventional similarity metric function was substituted with a perceptual similarity loss. Perceptual loss has been widely used and refined since it was proposed by J. Johnson et al. [37,38,39]. However, the pretrained VGG network, which is used in perceptual loss minimization, is implemented in 2D convolution and cannot be utilized directly to extract features of 3D images. Hence, we trained a 3D convolutional de-noising autoencoder (DAE) [40] network beforehand for the high-level feature extraction of CT and CBCT.

**Figure 6:**
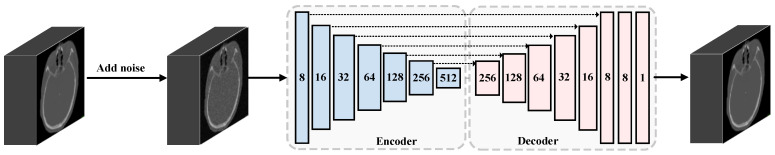
The details of the encoder–decoder architecture of a 3D convolutional de-noising auto-encoder (DAE) network with skip connections in between. It is trained to learn the representation of the input by restoring the original image without noise. *MSE* is chosen as the reconstruction loss. Once the DAE network is trained, the encoder can be used to extract the deep representation of the input.

**Figure 7:**
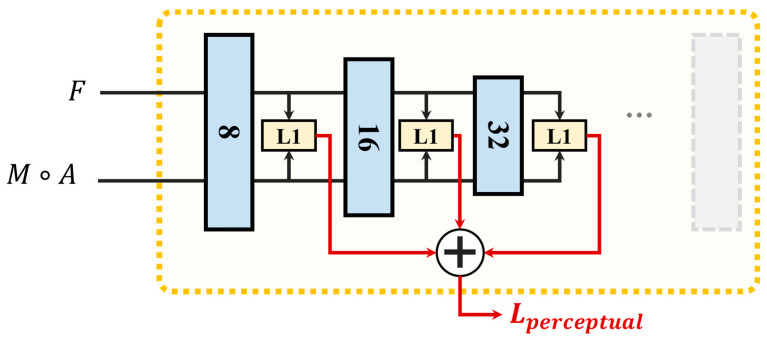
A transformed image and a fixed image are input into the perceptual similarity metric network. Multi-scale feature maps of the input images are extracted by the convolutional layers and are involved in the similarity evaluation. L1 represents the L1 loss.

The DAE learns the low dimensional representation of the noise-contaminated image through a CNN-based encoder, and then reconstructs the clean image by a decoder. A flowchart illustrating this process is shown in Figure 6. Before the original images are fed into the encoder path, they are augmented with noise. The encoder path is composed of a sequence of continuous convolution layers that extract and down-sample the feature maps until an intermediate feature vector of size (512,1) is obtained. Contrary to the encoder path, the decoder restores the resolution of the feature map in order to obtain noise-free images by deconvolution. The skip connection between the encoder and decoder fuses the multi-scale feature maps from the encoder and decoder to reduce the loss of image spatial information caused by down-sampling. By minimizing the mean square error between the output image and the original clean image, the encoder is trained to capture only the main features in the input data and ignore the impact of noise, allowing the convolution layers to learn to extract useful features. After the DAE network training is completed, the encoder with fixed weight is incorporated into the registration model to extract the features of CT and transformed CBCT images and calculate the distance between these deep features to assess the dissimilarity. The architecture of the proposed perceptual similarity metric network is shown in Figure 7. Three resolution levels of the feature maps are involved in the similarity loss calculation. *L1* loss is used to calculate the distance between the feature maps, the formula of perceptual similarity loss is as follows:

(7) $$L_{perceptual}(F,M(\phi ))=\sum_{i}^{N}\omega_{i}{\left\|R^{i}(F)-R^{i}(M(\phi ))\right\|}_{1},$$

where *N* denotes the number of feature resolution levels and is set to 3. $\omega_{i}$ is the weight of different scale feature differences. We empirically set $\omega_{1}=1$ and $\omega_{2}=\omega_{3}=0.5$. $R^{i}(F)$ and $R^{i}(M(\phi ))$ denote the feature maps of *F* and M(ϕ), respectively, from the *i*th layer of the metric network.

### 2.3. Anatomical Constraint Encoder Network

During the training process, an anatomically constrained encoder network is integrated in parallel with the primary registration network to enhance registration accuracy in the head region by leveraging corresponding tissues and organ segmentation masks. Two constraint terms are added to the loss function, which incorporates both pixel-level similarity and global content similarity computed on the transformed and fixed segmentation masks, respectively. This strategy is similar to the approach used in 2D image registration [34] and helps to enhance the global resemblance between segmentations.

The loss function at pixel-level similarity is calculated as follows:

(8) $$L_{dice}(S_{F},S_{M}(\phi ))=\frac{1}{K}\sum_{i\in [1,K]}\left(1-\frac{2(S_{F}^{i}\cap S_{M}^{i}(\phi ))}{\left|S_{F}^{i}|+|S_{M}^{i}(\phi )\right|}\right),$$

where *K* represents the number of anatomical structures.

It is apparent that $L_{dice}$ is calculated pixel by pixel, which may not provide the required global information to ensure a successful registration. As per the same process in Figure 6, we train the encoder of the DAE network in advance to learn a lower-dimensional representation of the anatomical segmentations. The encoder is responsible for learning the lower-dimensional representations that capture vital information about the input, such as shape and topology.

The acquired representations can be introduced into the registration model as prior knowledge. As shown in Figure 3b, the transformed anatomical segmentation and the fixed anatomical segmentation are input into the pretrained encoder, and the global similarity of the anatomical segmentation is evaluated by calculating the distance between the learned representations. We refer to the representations encoded by the anatomical constraint encoder as *ACE*(·). The formula is as follows:

(9) $$L_{ae}=||ACE(S_{F})-ACE(S_{M}(\phi ))|{|}_{2}^{2},$$

The final loss function of the network is as follows:

(10) $$L=L_{perceptual}+\lambda_{ae}L_{ae}+\lambda_{\mathrm{dice}}L_{\mathrm{dice}},$$

**Figure 8:**
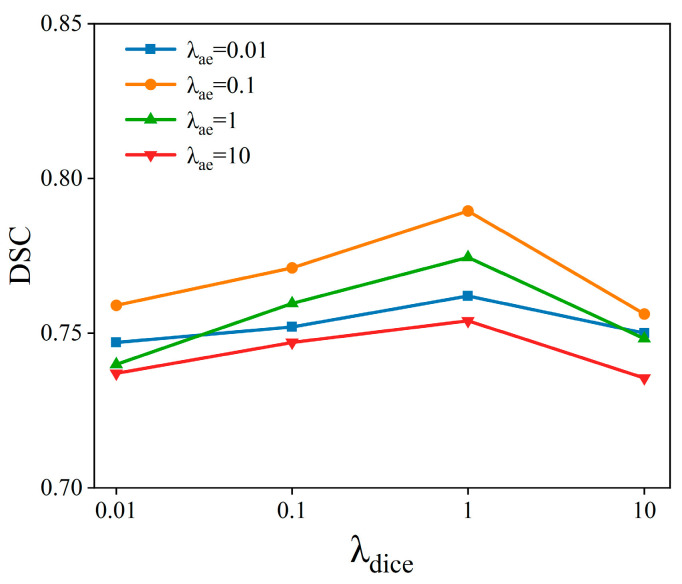
The results are produced by the ACSwinNet under different weighting parameters. The horizontal axis represents the different selections for hyperparameter *λ_dice_*, while the various colors of the lines in the graph indicate the different selections for another hyperparameter, *λ_ae_*. The vertical axis denotes the DSC values under these parameter settings.

We investigated two hyperparameters, *λ_dice_* and *λ_ae_*, and their values ranged from 0.01 to 10, changing in increments of tenfold. This means that we conducted a total of 16 different experiments with various combinations of these two hyperparameters. For each parameter combination, we assessed the model’s performance on the validation set to measure its performance. As shown in Figure 8, the best result is achieved when *λ_dice_* = 1 and *λ_ae_* = 0.1.

## 3. Experiments

### 3.1. Datasets and Pre-Processing

With the approval and consent of the Institutional Review Committee, we collected the planning head-neck CT image data and corresponding CBCT image data of 190 patients, with the datasets uploaded to Zenodo [41]. Planning CT images were acquired using the Brilliance CT Big Bore Scanner (Phillips Medical Systems, Andover, MA, USA). Each CT image dataset includes 90–176 slices with a thickness of 3 mm, and each slice has 512 × 512 voxels with a resolution of 1.1719 mm. Multiple CBCT images were acquired using an onboard imager (OBI, Varian Medical Systems, Palo Alto, CA, USA) in full-fan mode by Varian Edge linear accelerator during treatment, but only the images obtained on the first day of treatment were used in this study. Each CBCT data contains 88 slices with a thickness of 3 mm. Each slice has 270 × 270 voxels with a resolution of 1 mm. All the scans have ground truth segmentations, each of which contains five anatomical structures.

**Figure 9:**
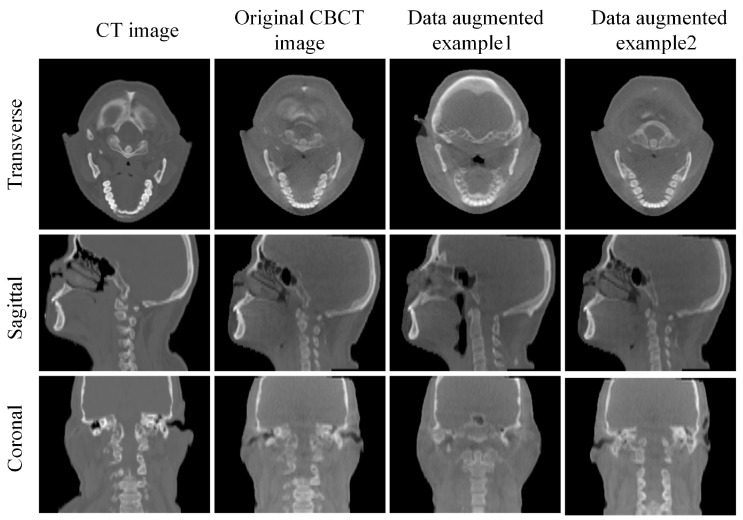
The visualization of the CT image, the original CBCT image, and examples of the original CBCT image after undergoing various rotational and translational data augmentations.

To keep the same spatial resolution between CT images and CBCT images, we resampled the CT image to a resolution of 1 mm × 1 mm × 3 mm. Both CT and CBCT images contain extraneous information, such as background regions, shoulder regions, and treatment beds. Thus, we cropped all CT and CBCT images to a standardized size and removed any irrelevant information within the field of view (FOV) to ensure that the images shared a consistent receptive field that covered only the head-neck region of interest. The final CT and CBCT images contain 240 × 240 × 64 voxels with a resolution of 1 mm × 1 mm × 3 mm. We split the dataset into 140, 10 and 40 for training, validation and test sets, respectively. To enhance the robustness of our model, we applied data augmentation in real time during model training. During training, the moving images in the training data will undergo random angle rotations ranging from −3 to +3 degrees, as well as translations of −3 to +3 pixels, Figure 9 illustrates examples of data augmentation. Therefore, for each training iteration, a pair of images with different initial misalignments is fed into the model. Additionally, we randomly selected five samples from the test set and applied the same operations to introduce larger misalignment, thus we obtained 45 image pairs for the test.

### 3.2. Baseline Methods

To examine and highlight the relative advantages of our proposed Swin Transformer-based registration method, we conducted a comparative study with two other distinct categories of registration techniques: conventional registration methods and CNN-based methods.

We conducted conventional rigid registration using ANTs toolkit [42]. Specifically, we utilized “Rigid” as the registration parameter and adaptive gradient descent as the optimization method, with mutual information as the similarity measure. In addition, we also included the modification of ANTs, denoted as ANT-Local in our study, where only the upper half of the images are utilized in the registration process. This modification simulates the clinical practice wherein doctors select the head region for registration to achieve higher accuracy in that specific region.

**Figure 10:**
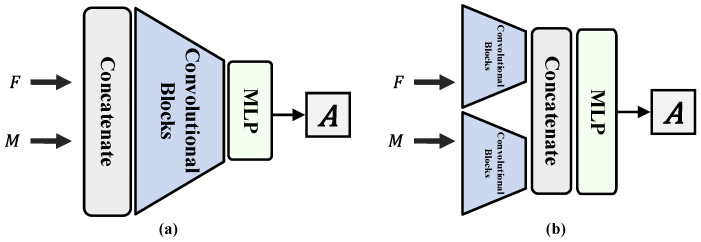
Two CNN-based rigid registration network architectures. (**a**) Images are concatenated before being fed into the network (VTN). (**b**) Image features are extracted separately and then concatenated (ConvNet).

To demonstrate the superiority of the Swin Transformer-based registration method over CNN-based registration methods, we incorporated two types of CNN-based registration methods in our experiments: image concatenation methods, such as VTN [43], which concatenate image pairs before feature extraction by convolution layers, and twin network types, such as ConvNet [44], which extract image features by convolution layers separately and then concatenate them on the channel dimension, as shown in Figure 10. For rigid registration, we modified ConvNet and VTN by changing their fully connected layer to output six parameters. We compared the registration performance between Swin Transformer and CNN by conducting ablation experiments, where we replaced the Swin Transformer part with these two convolution layers while keeping other structures and components unchanged to ensure a fair comparison. We denote the comparative networks used in the ablation experiments as AC-VTN and AC-ConvNet for the image concatenation method and twin network type, respectively. We also compare our method with the state-of-the-art Attention-Reg [22] method, which incorporates a cross-modal attention mechanism within its model architecture. Furthermore, the anatomical constraint encoder network is integrated into Attention-Reg to enhance focus on the head area. Each model was trained for 500 epochs to achieve optimal performance.

### 3.3. Measurement

The following metrics are used to quantitatively evaluate the registration results of the model: (i) The Dice similarity coefficient (DSC), which measures the degree of overlap between the anatomical segmentations of CT and CBCT images after registration. The DSC ranges between 0 and 1, with a higher value indicating better performance, and a value of 1 indicating complete overlap between the two segmentations. Five subcortical anatomical structures, including eyes, optic nerve, brainstem, spinal cord, and larynx, distributed in the head and neck regions, were segmented by experts for evaluation. After using the traditional elastic registration algorithm to elastically register the CT image to the CBCT, the segmentation results of the deformed CT image are used as the segmentation map for the CBCT image. (ii) Structural similarity (SSIM), which is independent of brightness and contrast, evaluates the similarity between the registered CBCT image and CT image structurally. A higher SSIM value indicates better performance. (iii) Target registration error (TRE), which calculates the mean distance of landmarks with anatomical significance on each pair of CT and CBCT images in the test set. A lower value means higher registration accuracy. The landmarks are manually marked by experts at the corresponding positions of CT and CBCT images from the test set, ensuring clear and anatomically significant positions that remain consistent across different patients. The head region and neck region of each pair of images are each marked with 10 landmarks. TRE_ H and TRE_ N represent the target registration error of the head region and neck region, respectively. In addition, the average time required for different methods to register a pair of images is also calculated.

### 3.4. Implementation Details

All deep learning-based registration methods in this study are implemented using Pytorch 2.4.0. We opt to use the Adam optimizer with a learning rate of 0.0001. Additionally, we set the batch size to 1. Our experiments are trained and tested on a workstation equipped with i9-10900k CPU, 32GB RAM and Nvidia GeForce RTX 3090 GPU. All models were trained for 500 epochs.

## 4. Results

### 4.1. Registration Performance Comparison

**Figure 11:**
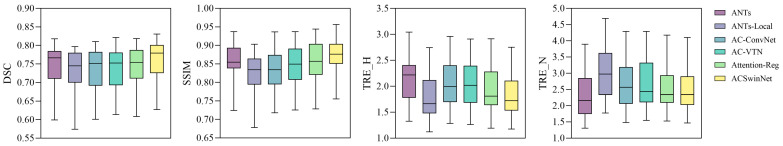
Box plots of different registration algorithms on different metrics.

Table 2 presents the average results of head-neck CT and CBCT images in the test set after registration by ANTs, ANTs-Local, VTN Rigid, ConvNet Rigid, Attention-Reg and ACSwinNet. We performed statistical evaluations through a t-test, with a significance level of *p* < 0.05 indicating a significant improvement. The results show that, before registration, there is a large misalignment in each test case, indicated by low average DSC and SSIM and high TRE value. However, after registration, there is a significant improvement in all the metrics, such as an increase in DSC from 0.39 to over 0.7 (*p* < 0.05). ACSwinNet only slightly outperforms the conventional rigid algorithm (implemented by ANTs) in terms of DSC and SSIM (*p* > 0.05), indicating that there is no significant difference between the methods in these metrics. For rigid registration, the overall potential for improvement in registration accuracy is limited. However, ACSwinNet significantly reduced the TRE of the head region (TRE_H) from 2.14 mm to 1.82 mm, compared with conventional method ANTs, with a statistically significant difference (*p* = 0.0011 < 0.05), which addresses the key issue tackled by this method. Additionally, the registration speed has increased by approximately 25 times. As for ANTs-Local, results show that the global registration accuracy of this method is not ideal. While the registration accuracy of the head region has been enhanced, with a TRE_H of 1.76 mm, the DSC remains only at 0.728. Additionally, the TRE of the neck region (TRE_N) has increased by over 29.2% (*p* = 0.0001 < 0.001) compared with ANTs. Attention-Reg achieves better performance than AC-VTN and AC-ConvNet, due to its cross-modal attention mechanism. Compared with the CNN-based methods AC-VTN, AC-ConvNet and Attention-Reg the Swin Transformer-based method ACSwinNet has better results in all of the metrics, suggesting that the Swin Transformer-based registration network in the present study has higher registration accuracy than the CNN-based registration network. Figure 11 illustrates box plots representing the performance of various registration algorithms across different evaluation metrics. From the figure, it can be observed that ACSwinNet receives favorable evaluations across all four metrics, while ANTs-Local performs best in TRE_H but exhibits the poorest performance in other metrics.

**Figure 12:**
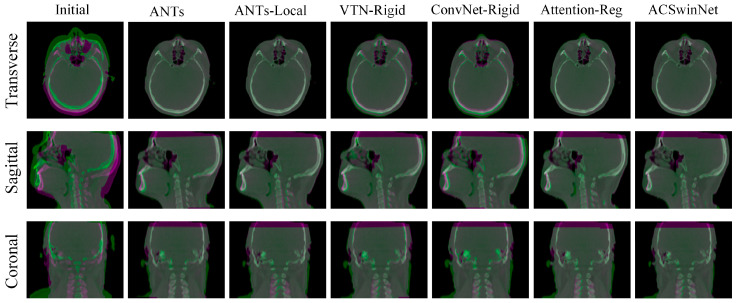
A visual comparison of image registration outcomes in a typical case of significant initial misalignment is presented. CT and CBCT images are color-coded in magenta and green, respectively, to aid visual discrimination, while the overlapped region appears gray. The results are displayed in transverse, sagittal, and coronal views. The first column represents the fusion image before registration, while the subsequent columns depict the fused images of outcomes produced by different techniques.

Figure 12 shows an example of a large misalignment between a CT and a CBCT image, with the channel fusion map displaying the registration results of various methods. The magenta and green regions represent CT and CBCT images, respectively, with gray indicating matched regions and magenta or green indicating misaligned regions. From the initial image, we can see that, before registration, the CT image and CBCT image are seriously misaligned, and that a large area of green or magenta regions can be observed. The registration results of AC-VTN and AC-ConvNet are shown in Figure 12 and there are still some green and magenta regions. This may be because the misalignment of the initial image is too large to be captured by the receptive field of CNN. In contrast, both the conventional algorithm ANTs and the proposed ACSwinNet show favorable registration results, with fewer magenta and green regions in the registered images.

### 4.2. Contribution of Anatomically Constrained Network

**Figure 13:**
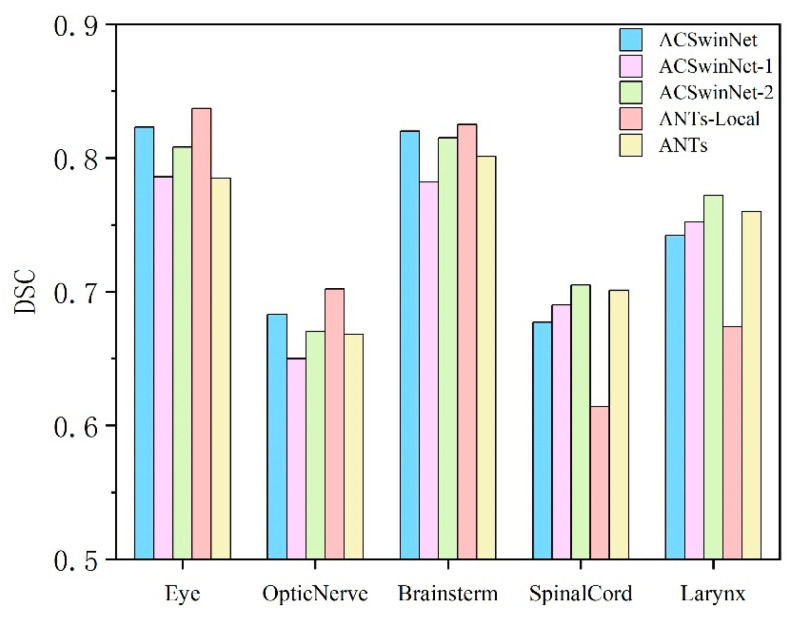
The bar plot illustrates the average DSC of the anatomical structures located in the head region and neck region. Notably, the eye, optic nerve, and brainstem are located in the head region, while the spinal cord and larynx are located in the neck region.

This section compares the head-neck registration methods ANTs, ANTs-Local, ACSwinNet and two variants of ACSwinNet, denoted as ACSwinNet-1 and ACSwinNet-2. Among these, ANTs-Local represents that only the local region (head region) of the image is selected for rigid registration using the ANTs method, which is a common registration strategy for doctors. ACSwinNet-1 does not use anatomical segmentations during training, while ACSwinNet-2 uses the segmentations from both the head region and the neck region during training. Table 3 shows that ACSwinNet-2 achieves the highest DSC of 0.759. However, as shown in Figure 13, ACSwinNet-2 has lower DSC for structures such as the eye, optic nerve and brain stem, as well as higher target registration errors in the head region compared with ACSwinNet (*p* = 0.0059 < 0.05). This may be due to the network not focusing on the registration of the head region after incorporating all of the anatomical segmentations. ACSwinNet-1 has a lower DSC on all structures than the method ACSwinNet-2, and reaches an average DSC of 0.732, which is also lower than ANTs. The TRE_H of ACSwinNet-1 is 2.26, which is significantly larger than that of ACSwinNet (*p* = 0.0002 < 0.001). These results suggest that the addition of anatomical segmentations can improve the registration accuracy of the model, and that the inclusion of anatomical segmentations for the specified region can significantly enhance the registration accuracy of the corresponding region in rigid registration.

The image registration results obtained using the ANTs-Local method exhibit the highest accuracy in the registration of the head region, as evidenced by the highest DSC achieved by the anatomical segmentations of the head region, including the eyes, optic nerves, and brainstem segmentations, as shown in Figure 13. Moreover, the target registration error in the head region (TRE_H) is also the lowest, measuring only 1.76 mm. However, this method performs poorly in the registration of the neck region, as indicated by the lowest DSC attained by the spinal cord and larynx segmentations in Figure 13. The target registration error in the neck region (TRE_N) is also higher than other methods, measuring 3.01 mm. On the other hand, the ACSwinNet method shows better DSC in the head region and TRE_H compared with the ANTs method, but slightly inferior DSC and TRE_N compared with ANTs, and much better than ANTs-Local in the neck region. Therefore, it can be concluded that the ACSwinNet method achieves a balance between ANTs and ANTs-Local, and effectively improves the accuracy of head registration while compromising only slightly on the accuracy of neck alignment.

**Figure 14:**
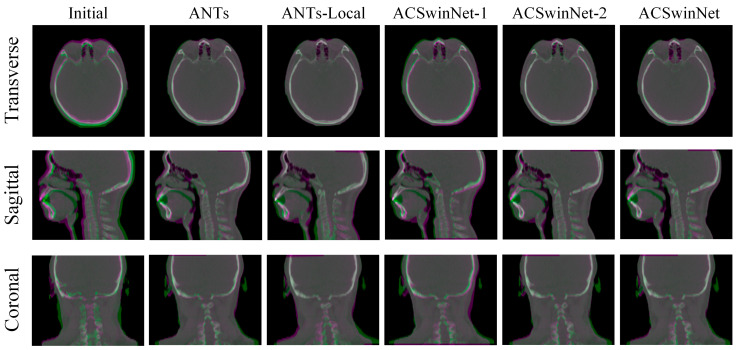
A visual comparison of the image registration result of images with slight non-linear deformation in the neck area is presented. For better observation, CT and CBCT images are colored in magenta and green, respectively, while the overlapping region is shown in gray. Results are displayed in the view of the transverse, sagittal and coronal sections. The first column shows the fused image before registration, whereas the other columns show the fused images of results produced by different methods.

Figure 14 demonstrates a comparison of various registration methods for images with neck deformation, using the registration results from a pair of images with significant displacement. The transverse planes of the results obtained by the ACSwinNet and ANTs-Local registration methods show a smaller colored area than those of other methods, indicating a higher alignment degree of the CT and CBCT images in the head region. ANTs and ACSwinNet-2 exhibit similar head registration accuracy, as seen from their transverse planes with a limited colored area. The global registration result is visible from the sagittal and coronal planes in Figure 14. The ANTs-Local method produces many colored areas in the neck region, suggesting poor registration accuracy. Although this method improves the registration accuracy of the head region, it may lead to poor registration accuracy of the neck region. The neck registration accuracy of ANTs, ACSwinNet-2, and ACSwinNet are relatively close, and all methods achieve satisfactory registration. However, ACSwinNet-1 performs poorly in both the head and neck regions without anatomical segmentations. The above observations highlight the way in which the proposed ACSwinNet method enhances the registration accuracy of the head region and ensures the registration accuracy of the neck region compared with the conventional method.

The comparison between ACSwinNet-1 and ACSwinNet results reveals the significant impact of anatomical segmentations on registration accuracy. Inspired by [34], we designed an anatomical constraint network to extract and analyze global information from anatomical segmentations. In addition, we incorporated two loss items, *L_ae_* and *L_dice_*, into the training process, which were computed using the transformed and fixed segmentation, respectively. These loss items were used to guide the model optimization and represent global content loss and pixel-level loss, respectively. Table 4 shows the registration performance of models with different anatomical loss items. The results indicate that the use of *L_ae_* and *L_dice_* alone can improve the model’s registration performance. The DSC of the model without any loss terms is only 0.732. However, incorporating different loss terms, such as *L_ae_* or *L_dice_*, leads to improved DSC values of 0.745 and 0.751, respectively. By incorporating both loss items into the loss function, the DSC increases to 0.745, and both TRE_H and TRE_N are significantly reduced. Thus, the model’s best performance is achieved when both *L_ae_* and *L_dice_* are utilized.

### 4.3. Contribution of Perceptual Similarity Metric Network

To demonstrate the superiority of the perceptual similarity metric network over commonly utilized metric functions in multimodal image registration, we compared the performance of the models trained with different metrics. SSIM, NCC and MI are chosen to be the metric functions in the present study, with the models trained with these metrics denoted as ACSwinNet-LSSIM, ACSwinNet-LNCC and ACSwinNet-LMI, respectively. The results shown in Table 5 indicate that the model trained with the perceptual similarity metric network, denoted as ACSwinNet-Lperceptual, achieves the best registration performance in terms of all indicators. Compared with ACSwinNet-LSSIM, ACSwinNet-LNCC and ACSwinNet-LMI, our method has improved the DSC by 0.016, 0.019, and 0.016, respectively. Furthermore, ACSwinNet-Lperceptual has also, to a certain extent, displayed improvements in SSIM and TRE of head-neck, thus illustrating that the perceptual similarity metric network can more effectively assess image similarity than conventional similarity metric functions.

**Figure 15:**
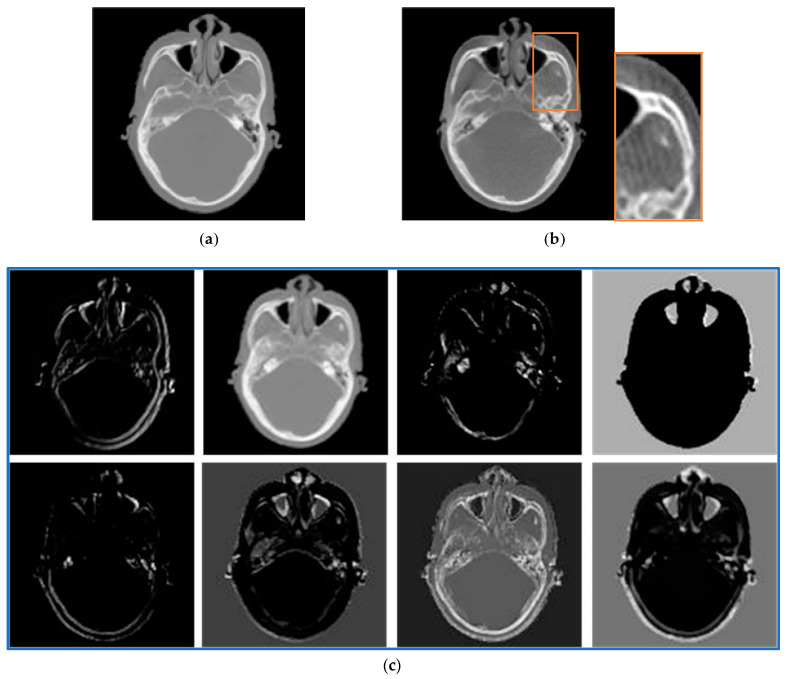

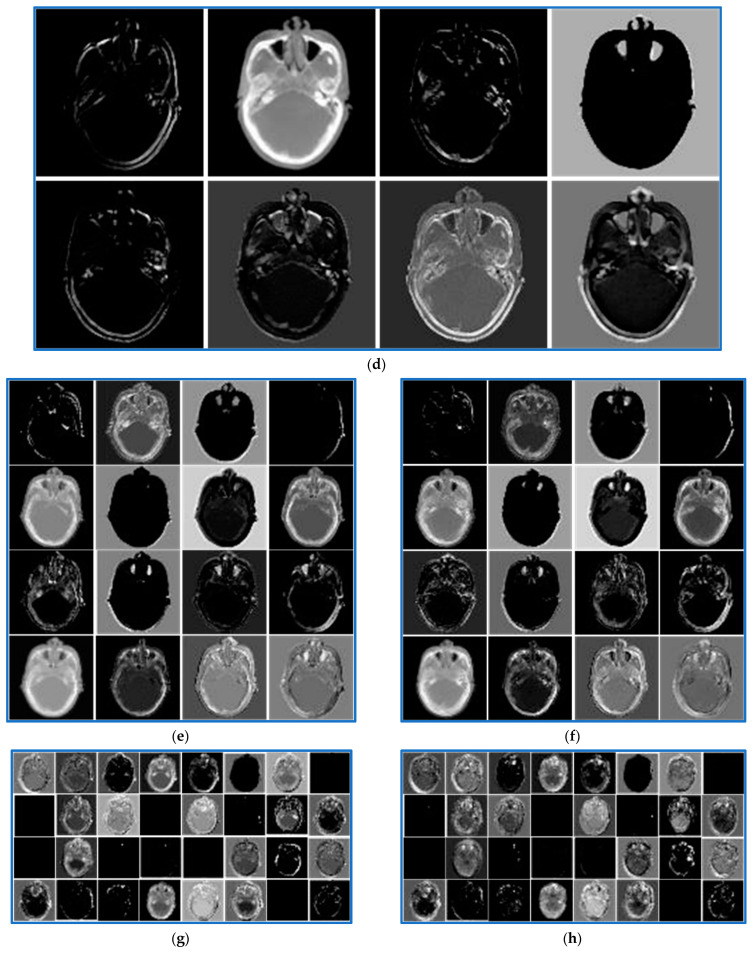
Slice examples of CT and CBCT feature maps at different scales. (**a**,**b**) The original image slices of CT and CBCT, respectively. (**c**–**h**) The feature maps at different resolution levels produced by the first three layers, with the resolution halved after each layer. Specifically, (**c**,**e**,**g**) are feature maps of CT images, while (**d**,**f**,**h**) are feature maps of CBCT images.

Figure 15 displays the multi-scale feature maps of CT and CBCT images that are extracted by the perceptual similarity metric network. The image shows that the CBCT image, which has lower quality, has severe artifacts that cannot identify the corresponding structures on the CT image. Consequently, this results in a decreased registration performance. Feature maps (b) and (c) demonstrate that the artifacts in the CBCT feature maps are less severe than those in the original image, and that, as the feature maps become deeper, there are fewer artifacts. This indicates that the pretrained convolution network can focus on the primary anatomical structure characteristics of the image and ignore the artifacts, effectively minimizing the impact of artifacts on the registration results. Moreover, computing the difference between the multi-scale CNN feature maps of multiple hidden layers can comprehensively evaluate the similarity between CT and CBCT images, and guide the learning process to accurately align the two images.

## 5. Discussion

The proposed method mainly aims to solve the problem that the registration accuracy requirement of the head region in head-neck CT-CBCT rigid registration is generally higher than that of the neck region, which is ignored by conventional methods. Visual and quantitative results illustrate that the anatomical constraint network with several segmentations from the head region can improve head region registration accuracy. This may be because the segmentation masks and loss items of the constraint encoder network impel the network to concentrate more on the head region. Our method is weakly supervised due to the use of extra anatomical segmentation information. In the experimental comparison, the performance of our method may be affected by the limited number of structures in the segmentation masks. We believe that including more anatomical structures in segmentation masks could potentially enhance the performance of the proposed method.

Given the large degree of initial misalignment between CT and CBCT images, we propose a Swin Transformer-based registration network to replace the CNN-based registration network, this would expand the field of view of the network in order to establish long-range dependencies through its self-attention mechanism, so that it can capture the misalignment information of images in a broader range. Owing to the broad receptive field, our method’s registration accuracy outperforms the CNN-based method. In addition, our method has the advantage of having a short running time compared with conventional methods and can be utilized to pre-align the target image pair in the deep-learning-based comprehensive registration framework by removing potential linear and large spatial misalignment.

As for the intensity differences between CT and CBCT images and artifacts in CBCT images, a perceptual similarity metric network is proposed for similarity evaluation by automatically extracting deep feature maps from multiple hidden layers, a method which has proven to be more effective than conventional metrics. Visual examination of the feature maps indicates a gradual elimination of artifacts in CBCT images. Because CNN is powerful in feature extraction, and the pretrained perceptual similarity metric network is not limited to a particular image type, it has the potential to be applied to the evaluation of similarity in other multimodal registrations.

Nonetheless, there is still room for improvement in our proposed method and experimental design. One significant limitation is the lack of publicly available head and neck CT-CBCT datasets for registration studies, which has constrained our testing to our private dataset. This limitation may hinder the generalizability of our findings, potentially affecting performance in real-world clinical practice. Future research should focus on validating the method with more diverse and publicly accessible datasets to ensure its broader applicability. Furthermore, training the model requires the acquisition of anatomical segmentation data for the relevant regions. However, manually annotated anatomical segmentation data are usually scarce in the medical field. In the future, such manually annotated data might be replaced by other types of prior information that are more readily accessible. Another challenge lies in generalizing the method to other different imaging modalities, when the disparities between images from different modalities are pronounced, such as between magnetic resonance images and ultrasound images, simple convolutional neural networks may fail to effectively extract corresponding feature information in perceptual loss. Therefore, it is essential to design more robust feature extraction methods. Therefore, we emphasize the need for further research to overcome these limitations and expand the method’s applicability across various contexts.

## 6. Conclusions

We propose ACSwinNet, a novel approach for head-neck CT-CBCT rigid registration that combines a Swin Transformer-based network, an anatomical constraint network and a perceptual similarity metric network. We propose to improve the head region registration accuracy by incorporating anatomical priors from the head region to the anatomical constraint network, which is guided by two loss items: pixel-level similarity *L_dice_* and global context *L_ae_*. We adopt the Swin Transformer-based network as the basic architecture due to its larger receptive field compared with CNN, which effectively handles the large misalignment between the image pair. In addition, we pretrained a 3D perceptual similarity metric network to evaluate the feature maps of the image pair at various scales from different hidden layers, resulting in improved discrimination and higher registration accuracy. Our comprehensive experiments on clinical data demonstrate that our method outperforms CNN-based methods in terms of overall accuracy on data with large initial misalignment. Furthermore, compared with conventional methods, our method achieves more accurate alignment in the head region in head-neck CT-CBCT registration and also speeds up the rigid registration process.

## Figures and Tables

**Table 1:** Overview of the related deep learning-based image registration methods.

Method	ROI	Modality	Architecture	Dimension	Transformation
C2FViT [29]	Brain	MR	Transformer	3D	Affine
ViT-V-Net [28]	Brain	MR	Transformer + CNN	3D	Deformable
VoxelMorph [33]	Brain	MR	CNN	3D	Deformable
AC-RegNet [34]	Lung	X-Ray	CNN	2D	Deformable
Attention-Reg [22]	Prostate	MR-TRUS	CNN	3D	Rigid/Deformable
InMIR-Net [19]	Brain/Nature	MR T1-T2	CNN	2D	Rigid/Deformable
MSReg [17]	Prostate	MR-TRUS	CNN	3D	Rigid
3DPose-Net [18]	Brain	MR	CNN	3D	Rigid
ACSwinNet (Ours)	Head&Neck	CT-CBCT	Swin Transformer + CNN	3D	Rigid

**Table 2:** Quantitative registration results (mean and standard deviation) of ANTs, ANTs-Local, AC-ConvNet, AC-VTN, Attention-Reg and our proposed method ACSwinNet. TRE_H and TRE_N represent the target registration error of the head region and neck region, respectively. ↑ means higher is better and ↓ means lower is better. The best results are highlighted in bold.

	DSC ↑	SSIM ↑	TRE_H (mm) ↓	TRE_N (mm) ↓	Time (s) ↓
Initial	0.390 (0.084)	0.651 (0.105)	8.74 (3.49)	10.28 (4.23)	/
ANTs	0.743 (0.051)	0.854 (0.044)	2.14 (0.45)	**2.33 (0.51)**	7.31
ANTs-Local	0.728 (0.062)	0.822 (0.058)	**1.76 (0.36)**	3.01 (0.83)	6.47
AC-ConvNet	0.734 (0.069)	0.830 (0.062)	2.07 (0.51)	2.67 (0.74)	**0.12**
AC-VTN	0.737 (0.058)	0.847 (0.057)	2.04 (0.50)	2.65 (0.68)	**0.12**
Attention-Reg	0.746 (0.055)	0.859 (0.056)	1.95 (0.44)	2.58 (0.62)	**0.16**
ACSwinNet	**0.755 (0.053)**	**0.870 (0.043)**	1.82 (0.39)	2.50 (0.60)	0.27

**Table 3:** Quantitative registration results (mean and standard deviation) of ANTs, ANTs-Local, our proposed ACSwinNet and its two variants ACSwinNet-1 and ACSwinNet-2. The target registration errors (TRE) of the head and neck regions are represented by TRE_H and TRE_N, respectively. ↑ means higher is better and ↓ means lower is better. The best results are highlighted in bold.

	DSC ↑	SSIM ↑	TRE_H (mm) ↓	TRE_N (mm)↓	Time (s) ↓
ANTs	0.743 (0.051)	0.854 (0.044)	2.14 (0.45)	2.33 (0.51)	7.31
ANTs-Local	0.728 (0.062)	0.822 (0.058)	**1.76 (0.36)**	3.01 (0.83)	6.47
ACSwinNet-1	0.732 (0.060)	0.829 (0.065)	2.26 (0.61)	2.49 (0.72)	**0.27**
ACSwinNet-2	**0.759 (0.049)**	**0.879 (0.041)**	2.08 (0.43)	**2.21 (0.52)**	**0.27**
ACSwinNet	0.755 (0.053)	0.870 (0.043)	1.82 (0.39)	2.50 (0.60)	**0.27**

**Table 4:** Quantitative registration results (mean and standard deviation) of ACSwinNet with different loss terms of the anatomical constraint network. TRE_H and TRE_N represent the target registration errors of the head region and neck region, respectively. ↑ means higher is better and ↓ means lower is better. The best results are highlighted in bold.

	**DSC ↑**	**SSIM ↑**	**TRE_H (mm) ↓**	**TRE_N (mm) ↓**	**Time (s) ↓**
ACSwinNet-1	0.732 (0.060)	0.829 (0.065)	2.26 (0.61)	**2.49 (0.72)**	**0.27**
ACSwinNet (*L_dice_*)	0.745 (0.059)	0.853 (0.050)	1.97 (0.51)	2.53 (0.65)	**0.27**
ACSwinNet (*L_ae_*)	0.751 (0.054)	0.861 (0.044)	1.87 (0.45)	2.54 (0.62)	**0.27**
ACSwinNet (*L_dice+ae_*)	**0.755 (0.053)**	**0.870 (0.043)**	**1.82 (0.39)**	2.50 (0.60)	**0.27**

**Table 5:** Quantitative registration results (mean and standard deviation) for the registration network with different similarity metric functions. TRE_H and TRE_N represent the target registration errors of the head region and neck region, respectively. ↑ means higher is better and ↓ means lower is better. The best results are highlighted in bold.

	DSC ↑	SSIM ↑	TRE_H (mm) ↓	TRE_N (mm) ↓	Time (s) ↓
ACSwinNet-L_SSIM_	0.739 (0.058)	0.860 (0.040)	2.05 (0.48)	2.55 (0.67)	**0.27**
ACSwinNet-L_NCC_	0.736 (0.059)	0.841 (0.056)	2.09 (0.53)	2.63 (0.72)	**0.27**
ACSwinNet-L_MI_	0.739 (0.055)	0.849 (0.042)	2.03 (0.50)	2.60 (0.64)	**0.27**
ACSwinNet-L_perceptual_	**0.755 (0.053)**	**0.870 (0.043)**	**1.82 (0.39)**	**2.50 (0.60)**	**0.27**

## Data Availability

The datasets used in the experiment have been uploaded to Zenodo and are openly available at https://zenodo.org/records/13231287 (accessed on 15 May 2024).
